# Microbes in Pool Filter Backwash as Evidence of the Need for Improved Swimmer Hygiene — Metro-Atlanta, Georgia, 2012

**Published:** 2013-05-17

**Authors:** Christopher Hutcheson,, Ryan Cira, Stanley L. Gaines, Kevin R. Jones, Walter Howard, David Hornsby, Maurice Redmond, Chris Rustin, Michele C. Hlavsa, Jennifer L. Murphy, Jothikumar Narayanan, Candace D. Miller, Brittany Cantrell, Vincent R. Hill, Michael J. Beach

**Affiliations:** Cobb & Douglas Public Health; DeKalb County Board of Health; Fulton County Dept of Health Svcs; Gwinnett County Health Dept; Georgia Dept of Public Health; Div of Foodborne, Waterborne, and Environmental Diseases, National Center for Emerging and Zoonotic Infectious Diseases

Filters physically remove contaminants, including microbes, from water in treated recreational water venues, such as pools. Because contaminants accumulate in filters, filter concentrates typically have a higher density of contamination than pool water. During the 2012 summer swimming season, filter concentrate samples were collected at metro-Atlanta public pools. Quantitative polymerase chain reaction (qPCR) assays were conducted to detect microbial nucleic acid. *Pseudomonas aeruginosa* was detected in 95 (59%) of 161 samples; detection indicates contamination from the environment (e.g., dirt), swimmers, or fomites (e.g., kickboards). *P. aeruginosa* detection underscores the need for vigilant pool cleaning, scrubbing, and water quality maintenance (e.g., disinfectant level and pH) to ensure that concentrations do not reach levels that negatively impact swimmer health. *Escherichia coli*, a fecal indicator, was detected in 93 (58%) samples; detection signifies that swimmers introduced fecal material into pool water. Fecal material can be introduced when it washes off of swimmers’ bodies or through a formed or diarrheal fecal incident in the water. The risk for pathogen transmission increases if swimmers introduce diarrheal feces. Although this study focused on microbial DNA in filters (not on illnesses), these findings indicate the need for swimmers to help prevent introduction of pathogens (e.g., taking a pre-swim shower and not swimming when ill with diarrhea), aquatics staff to maintain disinfectant level and pH according to public health standards to inactivate pathogens, and state and local environmental health specialists to enforce such standards.

During June–August 2012, county (Cobb, DeKalb, Fulton, and Gwinnett) and state environmental health specialists collaborated with CDC to collect filter concentrates at a convenience sample of public pools. The study protocol entailed collecting a 1-liter filter backwash* sample 30 seconds after the start of backwash flow and immediately neutralizing any free chlorine (the form of chlorine that inactivates pathogens), using 2.5 mL of a 10% sodium thiosulfate solution. Additionally, the following data were collected using a standardized form: type of filter media; pool location (i.e., indoor versus outdoor), setting (i.e., membership/club, municipal, or waterpark), and primary patron designation (i.e., adults and children versus primarily children); type of disinfectant used; visible signage instructing patrons not to swim when ill with diarrhea; estimated number of swimmers in the past week; and estimated number of days since last filter backwash. No pool identifiers were collected.

During December 2012–March 2013, nucleic acid was extracted from each sample (1), and qPCR assays (Table 1), were conducted to detect nucleic acid of *E. coli*, *P. aeruginosa*, *Giardia intestinalis*, *Cryptosporidium* spp., *E. coli* O157:H7 (a pathogenic toxin–producing *E. coli*), noroviruses GI and GII, and adenovirus.† Detection of a study microbe was defined as a qPCR cycle threshold§ value <40.

All but one of the pool filters in the study were rapid sand filters; the remaining filter used diatomaceous earth. At least one of the assayed microbes was detected in 121 (75%) of 161 filter backwash samples collected. *P. aeruginosa* was detected in 95 (59%) samples. *E. coli* was detected in 93 (58%) samples. *P. aeruginosa* and *E. coli* were both detected in 67 (42%) samples. *G. intestinalis* was detected in two samples. *Cryptosporidium* spp. were detected in one sample. Neither *E. coli* O157:H7, norovirus GI, norovirus GII, nor adenovirus was detected in any of the samples. The proportion of samples positive for *E. coli* significantly (p≤0.05) differed between membership/club and municipal pools (Table 2). The proportion of samples positive for *P. aeruginosa* significantly differed between venues treated with traditional chlorine products combined with ultraviolet light disinfection versus those treated with saltwater-generated free chlorine.¶ Most (71% [10 of 14]) pools with saltwater-generated free chlorine were located outdoors.

## Editorial Note

The detection of *E. coli* in over half of filter backwash samples indicates that swimmers frequently introduced fecal material into pools and thus might transmit infectious pathogens to others. The risk for transmission and recreational water illness (RWI)** increases if swimmers introduce feces when ill with diarrhea (Box). A single diarrheal contamination incident can introduce 10^7^–10^8^ *Cryptosporidium* oocysts (2) into the water, a quantity sufficient to cause infection if a mouthful of water from a typical pool is ingested (3). Additionally, each person has an average of 0.14 grams of fecal material on their perianal surface that could rinse into the water (4) if swimmers fail to take a pre-swim shower with soap. The 1) frequent occurrence of fecal contamination of pools documented in this study and 2) marked increase in the incidence of RWI outbreaks, which is driven by the substantially increasing incidence of acute gastrointestinal illness outbreaks associated with pools and caused by pathogens transmitted by the fecal-oral route (particularly the extremely chlorine-tolerant parasite, *Cryptosporidium*) (5), underscore the need for improved swimmer hygiene (e.g., taking a pre-swim shower and not swimming when ill with diarrhea). This study also found that the proportion of samples positive for *E. coli* significantly differed between membership/club and municipal pools. This finding might reflect differences in the number of swimmers who are either diapered children or children learning toileting skills.

Additionally, more than half of filter backwash samples were positive for *P. aeruginosa*. The detection of this ubiquitous microbe could reflect environmental (e.g., dirt or pool fill water), swimmer (e.g., fecal material or skin), or fomite (e.g., kickboards) contamination. Once in a pool, *P. aeruginosa* inhabits and amplifies in biofilms on moist or submerged surfaces, such as pool walls, plumbing, and filters. Further investigation is needed to better characterize *P. aeruginosa* contamination of pools and its contributing factors. *P. aeruginosa* can cause RWI (e.g., otitis externa or dermatitis) outbreaks when adequate disinfection is not consistently maintained (5). The proportion of samples positive for *P. aeruginosa* significantly differed between venues treated with traditional chlorine products combined with ultraviolet light disinfection versus those treated with saltwater-generated free chlorine. The reason for this association is unclear but might reflect differences in swimmers or pool location, age, or design. Pool operator vigilance (e.g., cleaning, scrubbing surfaces, and maintaining water quality [e.g., disinfectant level and pH]) and enforcement of such public health standards by state and local environmental health specialists can minimize *P. aeruginosa* amplification and thus prevent a negative impact on swimmer health.

BOX **Swimmer hygiene recommendations**

**Keep feces and urine out of the water.**

- Don’t swim when you have diarrhea.
- Shower with soap before you start swimming.
- Take a rinse shower before you get back into the water.
- Take bathroom breaks every 60 minutes.
- Wash your hands after using the toilet or changing diapers.

**Check the chlorine level and pH before getting into the water.**

- Pools: proper chlorine level (1–3 mg/L or parts per million) and pH (7.2–7.8) maximize pathogen inactivation.
- Most superstores, hardware stores, and pool-supply stores sell pool test strips.

**Don’t swallow the water you swim in.**

**Take some extra steps if you are the parent of a young child.**

- Take children on bathroom breaks every 60 minutes or check diapers every 30–60 minutes.
- Change diapers in the bathroom or diaper-changing area and not at poolside where pathogens can rinse into the water.

Additional information available at http://www.cdc.gov/healthyswimming.

The findings in this report are subject to at least four limitations. First, the pools sampled in this study are a convenience sample of pools in metro-Atlanta, and thus study findings cannot be generalized to pools in metro-Atlanta or beyond. However, the incidence of RWI outbreaks of acute gastrointestinal illness throughout the United States suggests that swimmers frequently introduce fecal material and pathogens into recreational water throughout the country. Second, qPCR results alone cannot be used to determine whether the detected pathogens were viable or infectious or determine the level of swimmer risk; qPCR detects viable microbes as well as those inactivated by disinfection. Of note, no RWI outbreaks associated with pools were detected in Georgia in 2012. Third, pool operators were asked to estimate the number of swimmers in the past week and number of days since last filter backwash; however, the data were deemed unreliable and thus could not be used to characterize the relationship between either of these factors and the detection of microbes in filter backwash samples. Finally, *E. coli* are found in fecal material from warm-blooded animals, not just humans. However, the *E. coli* detected in the pool filter backwash samples is most likely of human origin given that swimming is the most popular sport among children (6), over one third of the samples that tested positive for *E. coli* came from filters of indoor pools, and public outdoor pools are fenced in to limit access.

What is already known on this topic?

Since 1978, the incidence of recreational water illness (RWI) outbreaks of acute gastrointestinal illness has substantially increased, driving the marked increase in incidence of RWI outbreaks overall. A major contributing factor is poor swimmer hygiene (i.e., diarrheal incidents) in the implicated pools. A 2006 survey of metro-Atlanta public pools focused on the detection of chlorine-tolerant parasites, *Cryptosporidium* spp. and *Giardia* in filter backwash samples.

What is added by this report?

In this survey, pool filter backwash samples were collected at metro-Atlanta public pools during the 2012 summer swim season; qPCR assays were conducted to detect *Escherichia coli* (a fecal indicator), *Pseudomonas aeruginosa*, *Cryptosporidium* spp., *Giardia intestinalis, E. coli* O157:H7 (a pathogenic toxin–producing *E. coli*), norovirus genogroups I and II, and adenovirus. *E. coli* was detected in 93 (58%) of 161 samples collected. qPCR results alone cannot be used to determine whether the detected microbes were viable or infectious or determine the level of swimmer risk; qPCR detects viable microbes as well as those inactivated by disinfection.

What are the implications for public health practice?

The detection of *E. coli* in more than half of pool filter backwash samples indicates that swimmers frequently introduced fecal material into pools and thus might transmit pathogens to others through recreational water. RWI prevention will be optimized when swimmers minimize introduction of pathogens into the water by practicing good hygiene, aquatics staff maintain disinfectant level and pH according to state and local public health standards to inactivate pathogens, and state and local environmental health specialists enforce such standards.

Swimmers have the power and responsibility to decrease the risk for RWIs by practicing good hygiene. In addition to minimizing the amount of fecal material introduced into recreational water, good swimmer hygiene, through bathroom breaks every 60 minutes and taking a pre-swim shower, minimizes the amount of urine and sweat introduced into the water (Box). Nitrogen in urine and sweat depletes free chlorine by combining with it to form di- and tri-chloramines, which are volatile respiratory and ocular irritants; free chlorine alone, at CDC-recommended concentrations, is not an ocular irritant. This study and others indicate that swimmers frequently introduce fecal material, microbes, urine (7), sweat, and other contaminants (8) into recreational water. Another study suggests that disinfectant level and pH frequently are not properly maintained (9). Together, they all underscore the importance of a strong partnership among the swimming public, aquatics staff, and public health to prevent RWIs. RWI prevention will be optimized when swimmers minimize introduction of pathogens into the water by practicing good hygiene, aquatics staff maintain disinfectant level and pH according to state and local public health standards to inactivate pathogens, and state and local environmental health specialists enforce such standards. This critical partnership depends on maintaining robust state and local pool inspection programs (10) that provide leadership by enforcing public health standards and serving as a healthy swimming resource to aquatics staff and swimming public.

## Figures and Tables

**TABLE 1:** Target genes and molecular testing methodologies, by microbe — metro-Atlanta, Georgia, December 2012–March 2013

Microbe	Target gene	Molecular testing methodology
*Escherichia coli*	*uid A*	Sandhya S, Chen W, Mulchandani A. Molecular beacons: a real-time polymerase chain reaction assay for detecting *Escherichia coli* from fresh produce and water. Anal Chim Acta 2008;614:208–12.
*Pseudomonas aeruginosa*	*ecf*X	Amagliani G, Parlani ML, Brandi G, Sebastianelli G, Stocchi V, Schiavano GF. Molecular detection of *Pseudomonas aeruginosa* in recreational water. Int J Environ Health Res 2012;22:60–70.
*Giardia intestinalis*	18S rRNA	Manuscript submitted for publication.
*Cryptosporidium* spp.	18S rRNA	Jothikumar N, da Silva AJ, Moura I, Qvarnstrom Y, Hill VR. Detection and differentiation of *Cryptosporidium hominis* and *Cryptosporidium parvum* by dual TaqMan assays. J Med Microbiol 2008;57:1099–105.
*E. coli* O157:H7	*eae*	Sharma VK, Dean-Nystrom EA. Detection of enterohemorrhagic *E. coli* O157:H7 by using a multiplex real-time PCR assay for genes encoding intimin and Shiga toxins. Vet Microbiol 2003;93:247–60.
Noroviruses GI and GII	ORF1-ORF2	Hill VR, Mull B, Jothikumar N, Ferdinand K, Vinje J. Detection of GI and GII noroviruses in ground water using ultrafiltration and TaqMan real-time RT-PCR. Food Environ Virol 2010;2:218–24.
Adenovirus	Hexon	Jothikumar N, Cromeans TL, Hill VR, Lu X, Sobsey MD, Erdman DD. Quantitative real-time PCR assays for detection of human adenoviruses and identification of serotypes 40 and 41. Appl Environ Microbiol 2005;71:3131–6.

**TABLE 2:** Microbes in filter backwash samples from public pools (n = 161), by selected characteristics — metro-Atlanta, Georgia, 2012

Characteristic	Backwash samples qPCR-positive for *Pseudomonas aeruginosa* (n = 95)		p-value	Backwash samples qPCR-positive for *Escherichia coli* (n = 93)		p-value	Backwash samples qPCR-positive for any study microbes (n = 121)		p-value
		
	No.	(%)		No.	(%)		No.	(%)	
**Location**									
Indoor (n = 57)	28	(49)		33	(58)		39	(68)	
Outdoor (n = 104)	67	(64)	0.0591	60	(58)	0.9802	82	(79)	0.1432
**Setting**									
Membership/Club*† (n = 89)	55	(62)		44	(49)		64	(72)	
Municipal§ (n = 37)	22	(59)	0.8063	26	(70)	0.0321	30	(81)	0.2814
Waterpark¶ (n = 35)	18	(51)	0.2909	23	(66)	0.1017	27	(77)	0.5529
**Primary patron designation**									
Adults and children (n = 145)	85	(59)		81	(56)		106	(73)	
Primarily children (n = 15)	10	(67)	0.5458	11	(73)	0.1926	14	(93)	0.1181**
**Type of disinfectant**									
Chlorine (traditional), UV* (n = 21)	9	(43)		13	(62)		15	(71)	
Chlorine (traditional), ozone (n = 1)	1	(100)	NC	1	(100)	NC	1	(100)	NC
Chlorine (traditional) (n = 125)	74	(59)	0.1618	73	(58)	0.7626	94	(75)	0.7131
Chlorine (saltwater generated) (n = 14)	11	(79)	0.0365	6	(43)	0.2678	11	(79)	0.7115**
**Visible signage instructing patrons not to swim when ill with diarrhea**									
Yes (n = 35)	23	(66)		19	(54)		28	(80)	
No (n = 125)	72	(58)	0.3876	74	(59)	0.6025	93	(74)	0.4952

**Abbreviations:** qPCR = quantitative polymerase chain reaction; UV = ultraviolet light disinfection; NC = not calculated because of limited data.Acknowledgment

* Referent group.

† Membership/Club: any venue with limited access (e.g., apartment complexes and health and fitness centers).

§ Municipal: any city- or county-owned venue not classified as a waterpark.

¶ Waterpark: any venue with interactive water features, shallow-depth pool, or spray feature.

** Two-sided Fisher’s exact test used because 25% of the cells have expected counts <5. Otherwise, chi-square test was used.

## Footnotes

* Contaminants accumulate in pool filters, leading to a decrease in water flow through the filter. Consequently, filters need to be regularly backwashed. Backwashing reverses the direction of the flow of water so that contaminants trapped by the filter are dislodged and discharged to waste.

† CDC does not recommend testing the water or the filter backwash of treated recreational water venues (e.g., pools and hot tubs/spas) for microbes unless the venue is at least suspected to be associated with a waterborne disease outbreak. Maintaining proper disinfectant level and pH should prevent transmission of chlorine-susceptible pathogens.

§ Cycle threshold value is the fractional cycle number reported by real-time PCR instruments indicating the point at which the fluorescence associated with a positive DNA amplification reaction increases beyond the threshold associated with negative reactions.

¶ In saltwater pools, an electric current is passed through the water to generate free chlorine from sodium chloride. This free chlorine is the same as the free chlorine generated when traditional chlorine products are added to pool water.

** RWIs are caused by infectious pathogens transmitted by ingesting, inhaling aerosols of, or having contact with contaminated water in swimming pools, hot tubs/spas, water parks, interactive fountains, lakes, rivers, and oceans. RWIs also can be caused by chemicals in the water or chemicals that volatilize from the water and cause indoor air quality problems.
